# HIV-1 Envelope Glycoprotein Cell Surface Localization Is Associated with Antibody-Induced Internalization

**DOI:** 10.3390/v13101953

**Published:** 2021-09-29

**Authors:** Sai Priya Anand, Jérémie Prévost, Jade Descôteaux-Dinelle, Jonathan Richard, Dung N. Nguyen, Halima Medjahed, Hung-Ching Chen, Amos B. Smith, Marzena Pazgier, Andrés Finzi

**Affiliations:** 1 Centre de Recherche du CHUM, Montreal, QC H2X 0A9, Canada; sai.anand@mail.mcgill.ca (S.P.A.); jeremie.prevost@umontreal.ca (J.P.); jade.descoteaux-dinelle@umontreal.ca (J.D.-D.); jonathan.richard.1@umontreal.ca (J.R.); halima.medjahed.chum@ssss.gouv.qc.ca (H.M.); 2 Department of Microbiology and Immunology, McGill University, Montreal, QC H3A 2B4, Canada; 3 Département de Microbiologie, Infectiologie et Immunologie, Université de Montréal, Montreal, QC H2X 0A9, Canada; 4 Infectious Disease Division, Department of Medicine, Uniformed Services University of the Health Sciences, Bethesda, MD 20814-4712, USA; dung.nguyen.ctr@usuhs.edu (D.N.N.); marzena.pazgier@usuhs.edu (M.P.); 5 Department of Chemistry, University of Pennsylvania, Philadelphia, PA 19104, USA; chenhc@sas.upenn.edu (H.-C.C.); smithab@sas.upenn.edu (A.B.S.III)

**Keywords:** HIV-1, non-neutralizing antibodies, broadly neutralizing antibodies, Env, internalization, endocytosis, Env conformation, CD4, lipid microdomains

## Abstract

To minimize immune responses against infected cells, HIV-1 has evolved different mechanisms to limit the surface expression of its envelope glycoproteins (Env). Recent observations suggest that the binding of certain broadly neutralizing antibodies (bNAbs) targeting the ‘closed’ conformation of Env induces its internalization. On the other hand, non-neutralizing antibodies (nNAbs) that preferentially target Env in its ‘open’ conformation, remain bound to Env on the cell surface for longer periods of time. In this study, we attempt to better understand the underlying mechanisms behind the differential rates of antibody-mediated Env internalization. We demonstrate that ‘forcing’ open Env using CD4 mimetics allows for nNAb binding and results in similar rates of Env internalization as those observed upon the bNAb binding. Moreover, we can identify distinct populations of Env that are differentially targeted by Abs that mediate faster rates of internalization, suggesting that the mechanism of antibody-induced Env internalization partially depends on the localization of Env on the cell surface.

## 1. Introduction

Envelope glycoproteins (Env) of the human immunodeficiency virus (HIV-1) have long C-terminal cytoplasmic tails containing specific trafficking signals [1,2]. These allow for the endocytosis of Env from the surface of infected cells, which has been suggested to be a mechanism in place to minimize recognition by the host immune system. Mutations of these motifs have been shown to result in increased cell surface expression of Env and to correlate with increased Fc-mediated effector responses, such as antibody-dependent cellular cytotoxicity (ADCC), against infected cells [3]. Additionally, we have recently reported that the binding of broadly neutralizing antibodies (bNAbs) to Env accelerates its internalization from the surface of infected cells [4]. On the contrary, the binding of non-neutralizing antibodies (nNAbs) induced Env internalization at a significantly slower rate, allowing Env to remain on the cell surface for a prolonged period [4]. This phenomenon has also been observed with other retroviral glycoproteins, including the murine leukemia virus (MLV), where the binding of certain antibodies initiates signaling cascades within the cell, leading to cellular activation and enhancement of envelope glycoprotein internalization [5]. Furthermore, we also observed that upon dynamin inhibition, antibody-mediated Env internalization is significantly reduced and the susceptibility of infected cells to ADCC responses mediated by bNAbs increased [4]. Similarly, recent studies have also demonstrated enhancement of ADCC responses against human tumors upon temporary endocytosis inhibition [6]. Thus, antibody-induced antigen internalization from the cell surface decreases the overall recognition and elimination of target cells by effector cells.

In this study, we attempt to better understand the mechanisms by which bNAbs induce Env internalization and nNAb-bound Env persists on the cell surface for longer periods of time. Recent studies have highlighted the presence of different Env populations at the cell surface due to differential processing during its trafficking [7,8,9]. To investigate whether the differences in antibody-mediated Env internalization is due to distinct populations of Env on the cell surface being targeted, we utilize ligands such as small CD4-mimetics (CD4mc) and soluble CD4 (sCD4) to ‘force’ open the otherwise ‘closed’ Env populations and evaluate the rates of nNAb-mediated internalization. Our observations indicate that in addition to the conformation of Env and epitope availability, Env internalization could also depend on its localization on the cell surface.

## 2. Material and Methods

### 2.1. Ethics Statement

Written informed consent was obtained from all study participants (the Montreal Primary HIV Infection Cohort) [10,11]. Research adhered to the ethical guidelines of CRCHUM and was reviewed and approved by the CRCHUM institutional review board (ethics committee, approval number CE16.164-CA). Research adhered to the standards indicated by the Declaration of Helsinki. All participants were adult and provided informed written consent prior to enrolment in accordance with Institutional Review Board approval.

### 2.2. Cell Lines and Primary Cells

First, 293T human embryonic kidney cells (obtained from ATCC, Manassas, VA, USA) were cultured at 37 °C under 5% CO_2_ in Dulbecco’s modified Eagle’s medium (Wisent) containing 5% fetal bovine serum (VWR, Radnor, PA, USA) and 100 µg/mL of penicillin-streptomycin (Wisent, St. Bruno, QC, Canada). Primary CD4+ T lymphocytes were purified from resting PBMCs by negative selection and activated as previously described [12,13]. Briefly, PBMC were obtained by leukapheresis. CD4+ T lymphocytes were purified using immunomagnetic beads as per the manufacturer’s instructions (StemCell Technologies, Vancouver, BC, Canada). CD4+ T lymphocytes were activated with phytohemagglutinin-L (PHA-L; 10 µg/mL) for 48 h and then maintained in RPMI 1640 (Gibco, Waltham, MA, USA) complete medium supplemented with rIL-2 (100 U/mL).

### 2.3. Plasmids and Proviral Constructs

The vesicular stomatitis virus G (VSV-G)-encoding plasmid (pSVCMV-IN-VSV-G) was previously described [14]. The infectious molecular clone (IMC) of the transmitted/founder (T/F) virus CH58 was inferred and constructed as previously described [15,16]. The CH58 IMC with the L193A change in the Env glycoprotein (L193A) or defective for Nef and Vpu expression (Nef-Vpu-) were described elsewhere [17,18]. The JRFL IMC was also previously reported [19]. Plasmids used to transfect 293T cells include the pcDNA3.1 vector expressing the codon-optimized HIV-1 JRFL envelope glycoproteins and the pcDNA3.1 human CD4 expressor [20,21].

### 2.4. Viral Production, Infections, and Ex Vivo Amplification

To ensure similar levels of infection between viruses, vesicular stomatitis viruses G (VSVG)-pseudotyped viruses were produced and titrated as described [13]. Viruses were used to infect activated primary CD4 T cells from healthy HIV-1 negative donors by spin infection at 800× *g* for 1 h in 96-well plates at 25 °C. To expand endogenously infected CD4+ T cells, primary CD4+ T cells obtained from six antiretroviral therapy (ART)-treated HIV-1-infected individuals were isolated from PBMCs by negative selection. Purified CD4+ T cells were activated with PHA-L at 10 μg /mL for 48 h and then cultured for at least 6 days in RPMI-1640 complete medium supplemented with rIL-2 (100 U/mL).

### 2.5. Antibodies and Reagents

Anti-HIV-1 gp120 mAbs recognizing CD4-induced epitopes (19b, 17b; obtained from NIH AIDS Reagent Program), the outer domain (2G12; obtained from NIH AIDS Reagent Program, Manassas, VA, USA) and the gp120-gp41 interface (PGT151; obtained from IAVI, New York, NY, USA) were used for cell surface staining of infected cells. Additionally, the following constructs were also used for cell surface staining: 17b-sCD4 and 19b-sCD4 constructs, which are hybrid proteins and consist of anti-gp120 Abs linked to the C-terminus of soluble CD4 (sCD4; D1D2 domains) via a flexible linker on each heavy chain [22]. Goat anti-human IgG Alexa Fluor 647 secondary Ab (Thermo Fisher Scientific, Waltham, MA, USA) was used to determine overall antibody binding and AquaVivid (Thermo Fisher Scientific) as a viability dye. The sCD4 protein was produced and purified as previously described [23]. The small molecule CD4 mimetic compound (CD4mc) BNM-III-170 were synthesized as described previously [24,25]. The CD4mc was analyzed, dissolved in dimethyl sulfoxide (DMSO) at a stock concentration of 10 mM, aliquoted, and stored at −80 °C until further use. For Western blot analyses, a mouse anti-CD71 monoclonal antibody (clone OKT-9; Thermo Fisher Scientific) was used as a control for detergent soluble membranes (DSM) and was followed by incubation with a horseradish peroxidase (HRP)-conjugated antibody specific for the Fc region of mouse IgG (Thermo Fisher Scientific). An HRP-conjugated cholera toxin subunit B (CTx-B) (Invitrogen Rockford, IL, USA) was used to detect ganglioside GM1 as a control for detergent-resistant membranes (DRM), and HRP-conjugated streptavidin (Thermo Fisher Scientific) was used to detect cell surface biotinylated Env. For confocal microscopy analyses, DAPI (Sigma, St. Louis, MO, USA) was used for nucleic acid staining. Antibodies 19b and 17b were conjugated with Alexa Fluor 647 and 594 probes (Thermo Fisher Scientific), respectively, as per the manufacturer’s protocol and used for confocal microscopy analyses. Alternatively, staining with 17b-sCD4 was performed in combination with the goat anti-human IgG Alexa Fluor 594 secondary Ab (Thermo Fisher Scientific).

### 2.6. Antibody-Induced Internalization Assay by Flow Cytometry

At 48 h post-infection, HIV-1-infected primary CD4+ T cells were incubated with 5 μg/mL of anti-Env antibodies or 10 μg/mL of 19b-sCD4 or 17b-sCD4 chimeric protein for 30 min at room temperature to allow for antibody attachment. To allow nnAbs binding, either sCD4 (10 μg/mL) or the CD4-mimetic compound BNM-III-170 (5 μM) were also added to the cells. To remove unbound antibodies, cells were washed three times with cold phosphate-buffered saline (PBS). This was followed by incubation at 37 °C to start the internalization process. After different time points, cells were fixed with 2% paraformaldehyde (PFA) in PBS. To visualize remaining antigen-antibody complexes on the cell surface, cells were stained with a goat anti-human conjugated with Alexa Fluor 647 secondary Ab (Thermo Fisher Scientific). Some cells were fixed after the primary incubation with the anti-Env antibodies as a control (time point 0 min). Dead cells were excluded using the live/dead fixable AquaVivid stain (Thermo Fisher Scientific). The reduction in surface expression for a given time point was normalized by using the following equation:

((Mean Fluorescence Intensity at X min)/(Mean Fluorescence Intensity at 0 min)) × 100

HIV-1-infected cells were identified by intracellular staining of HIV-1 p24 using the Cytofix/Cytoperm Fixation/Permeabilization Kit (BD Biosciences Mississauga, ON, Canada) and the PE-conjugated anti-p24 mAb (clone KC57; Beckman Coulter, Brea, CA, USA). The percentage of infected cells (p24+) was determined by gating the living cell population based on viability dye staining with AquaVivid (Thermo Fisher Scientific). Samples were analyzed on an LSRII cytometer (BD Biosciences), and data analysis was performed using FlowJo v10.7.2 (Tree Star, Ashland, OR, USA).

### 2.7. Antibody-Induced Internalization Assay by Confocal Microscopy

For confocal microscopy analyses, 293T cells were plated in poly-D-lysine coated 14 mm MatTek (Ashland, MA, USA) dishes with #0 coverslip bottoms. The 293T cells were transfected with 1 μg JRFL Env (codon-optimized) plasmid with or without 1 μg human CD4 plasmid. At 48 h post transfection, cells were incubated with prelabeled anti-gp120 antibodies (5 µg/mL) (+/− 5 μM of BNM-III-170) or a mix of 17b-sCD4 (10 µg/mL) and pre-coupled anti-human IgG (1:1000 dilution) in fresh media for 20 min, washed twice with PBS + 0.5% BSA, and incubated for the indicated amount of time at 37 °C to start the internalization process. After different time points, cells were fixed with PBS + 4% PFA for 30 min and then placed in PBS prior to imaging. All high-resolution images were obtained using a Zeiss AxioObserver Z1 Yokogawa CSU-X1 Spinning disk confocal microscope equipped with Piezo objectives, an Evolve EMCCD (512 × 512, 16 bit) monochrome camera (Photometrics, Tucson, AZ, USA), and 405-, 488-, and 561-nm lasers. Images were analyzed manually using ImageJ [26]. Briefly, regions of interest were drawn around entire cells and cytoplasmic regions. Total fluorescence for regions of interest were calculated as the area × (mean–minimum). Surface fluorescence was calculated as the total fluorescence minus the cytoplasmic fluorescence. Values are represented as surface/total ratios, normalized to the 0-min time point. The average intensity and area were measured and used to calculate the total fluorescence and the cytoplasmic fluorescence. The minimum intensity was subtracted from the mean intensity to correct for cytoplasmic background fluorescence.

### 2.8. Biochemical Isolation of Lipid Microdomains and Western Blot Analysis

At 48 h post-transfection, Env-expressing 293T cells (2 × 10^7^ to 4 × 10^7^) were washed twice with ice-cold PBS and cell surface proteins were biotinylated using EZ-Link™ Sulfo-NHS-LC-Biotin (Thermo Fisher Scientific) as per the manufacturer’s protocol for 30 min at 4 °C. Cells were incubated with 10 μg/mL of respective anti-Env antibodies for cell surface staining for 30 min at room temperature. Cells were then lysed on ice for 30 min in 1 mL of 1% Triton X-100 TNE lysis buffer (25 mM Tris (pH 7.5), 150 mM NaCl, 5 mM EDTA) supplemented with protease inhibitor cocktail (Thermo Fisher Scientific). The cell lysates were homogenized with a tissue grinder (Fisher, Hampton, NH, USA) and centrifuged for 5 min at 720× *g* at 4 °C in a microcentrifuge. The supernatant was mixed with 1 mL of 80% sucrose in TNE lysis buffer, placed at the bottoms of ultracentrifuge tubes, and overlaid with 6 mL of 30% and 3 mL of 5% sucrose in TNE lysis buffer. The lysates were ultracentrifuged at 4 °C in a TH641 rotor (Thermo Fisher Scientific) for 16 h at 38,000 rpm. After centrifugation, the Triton X-100-insoluble, low-density material was visible as a band migrating on the boundary between 5 and 30% sucrose. One 4 mL and two 3 mL fractions were collected from the top; 100μL of each fraction was analyzed immediately by Western blotting and the rest was used for immunoprecipitation. Precipitation antibody-bound biotinylated cell surface JRFL envelope glycoproteins from cell lysates were performed for 1 h at 4 °C in the presence of 50 μL of 10% protein A-Sepharose (Cytiva, Marlborough, MA, USA). Aliquots of sucrose gradient fractions were analyzed by sodium dodecyl sulfate-polyacrylamide gel electrophoresis (SDS-PAGE) on 10% polyacrylamide gels. The proteins were transferred to supported nitrocellulose membranes and probed with either HRP-conjugated streptavidin (1:2500), anti-CD71 (1:1000) followed by HRP-conjugated anti-mouse IgG (1:10,000) or HRP-conjugated CTx-B (200 ng/mL). HRP enzyme activity was determined after the addition of a 1:1 mix of Western Lightning oxidizing and luminol reagents (PerkinElmer Life Sciences, Waltham, MA, USA).

### 2.9. Statistical Analyses

Statistics were analyzed using GraphPad Prism version 9.1.1 (GraphPad, San Diego, CA, USA). Every data set was tested for statistical normality, and this information was used to apply the appropriate (parametric or nonparametric) statistical test. Any *p* values of < 0.05 were considered significant; significance values are indicated as * *p* < 0.05, ** *p* < 0.01, *** *p* < 0.001, **** *p* < 0.0001.

## 3. Results and Discussion

### 3.1. HIV-1 Env ‘Opening’ Accelerates Its Antibody-Mediated Internalization from the Cell Surface

**Figure 1:**
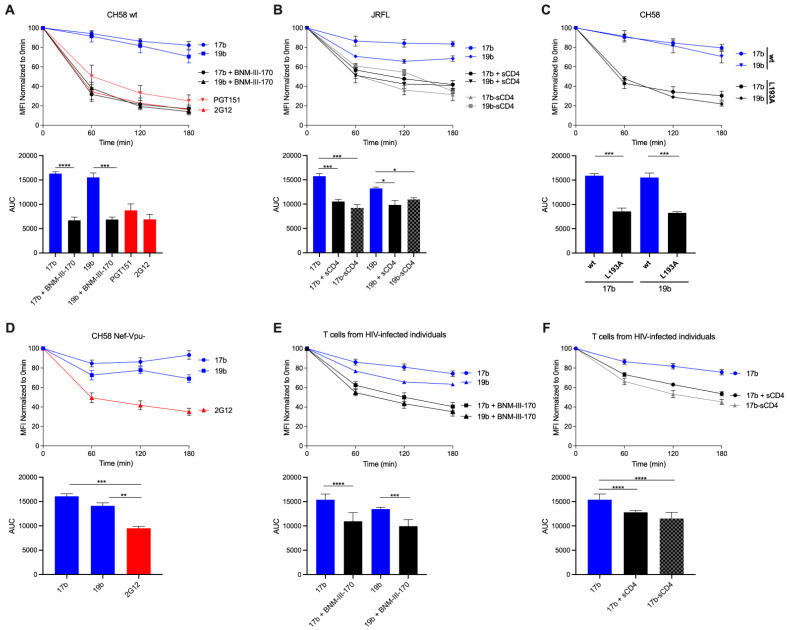
Antibody-internalization of HIV-1 Env from the cell surface can be accelerated upon the selective opening of Env. (**A**–**D**) Cell surface staining of primary CD4+ T cells infected in vitro with (**A**) CH58 T/F virus, (**B**) JRFL WT virus, (**C**) CH58 T/F WT or L193A virus, and (**D**) CH58 T/F virus defective for Nef and Vpu expression was performed 48 h post-infection. (**E**,**F**) Primary CD4+ T cells from at least three different HIV-1-infected individuals were isolated and reactivated with PHA-L for 48 h, followed by incubation with IL-2 to expand the endogenous virus. Cell surface staining of endogenously infected primary CD4+ T cells was performed upon reactivation. (**A**–**F**) Antibody binding was detected using Alexa Fluor 647-conjugated anti-human secondary Abs. (Top) Quantification of remaining antibody–Env complexes on the cell surface over different timepoints is expressed as percentage of the MFI relative to the 0 min timepoint control. (Bottom) Areas under the curve (AUC) were calculated based on MFI data sets using GraphPad Prism software. Error bars indicate means ± the SEM. Statistical significance was tested using an unpaired *t* test or a Mann-Whitney U test based on statistical normality (*, *p* < 0.05; **, *p* < 0.01; ***, *p*  <  0.001; ****, *p*  <  0.0001).

We first determined the rate of cell surface Env internalization upon the addition of certain nNAbs, the coreceptor binding site antibody 17b and the V3 crown antibody 19b, from the surface of primary CD4+ T cells infected with the transmitted/founder (T/F) virus CH58. As in our previous observations [4], the binding of nNAbs to surface Env remained steady over the course of 3 h at 37 °C and antibody-bound Env levels only reduced by ∼20% (Figure 1A). Upon the addition of a CD4mc, BNM-III-170, Env adopts its downstream CD4-bound conformation [27]. This allows for enhanced nNAb binding that preferentially recognize epitopes normally hidden in the ‘closed’ Env, including enhanced 17b and 19b binding [12,28,29]. In the presence of BNM-III-170, surface levels of 17b- and 19b-bound Env significantly declined by ~80% after 3 h, at a similar rate as the bNAb PGT151, indicating Env internalization (Figure 1A).

We confirmed these observations with soluble CD4 (sCD4), a version of the CD4 receptor lacking its transmembrane region. In this condition, sCD4 interacts with Env in *trans* and we observed faster rates of nNAb-induced Env internalization (Figure 1B). Furthermore, we also used Ab-sCD4 hybrid proteins which are designed to harbor two sCD4 molecules linked to a single antibody of interest [22]. Using 17b-sCD4 and 19b-sCD4 hybrids, surface levels of Env significantly declined by ~60% from the surface of primary CD4+ T cells infected with the JRFL virus (Figure 1B). Using other methods to selectively ‘open up’ the Env independently of CD4, we introduced the L193A substitution in Env, a mutation known to stabilize Env downstream conformations [18,30]. In a similar fashion as with the addition of BNM-III-170, surface levels of 17b- and 19b-bound Env declined by ~80% over 3 h at 37 °C using cells infected with the CH58 L193A virus (Figure 1C).

Interestingly, the deletion of Nef and Vpu, which impairs the capacity of the virus to downregulate CD4 resulting in Env sampling its ‘open’ conformation, [13] significantly slowed down the rate of 17b- and 19b-mediated Env internalization (Figure 1D) [4] when compared to the accelerated rates seen in the presence of BNM-III-170 in Figure 1A. Thus, these results suggest that ‘opening’ the Env is not sufficient to induce its internalization since sCD4, but not membrane-anchored CD4 (mCD4) are able to mediate nNAb-induced internalization. Whether this phenotype is related to the mode of interaction being in *trans* for sCD4 versus in *cis* for mCD4 [13] remains to be determined. Hypothetically, endogenous CD4 expression might result in Env-CD4 complex co-trafficking and the redirection of Env to different domains at the cell surface that are refractory to antibody-induced internalization.

We further confirmed our observations of antibody-mediated internalization upon the selective opening of Env with ex vivo-expanded endogenously infected CD4+ T cells. Primary CD4+ T cells were isolated from antiretroviral therapy (ART)-treated HIV-1-infected individuals and activated with PHA-L/IL-2, where viral replication was followed by intracellular p24 staining. In agreement with the results obtained with CH58 T/F and JRFL-infected primary CD4+ T cells, the binding of 19b and 17b induces Env internalization from the surface of endogenously infected primary CD4+ T cells in the presence of BNM-III-170 (Figure 1E), sCD4, or when using an Ab-sCD4 hybrid protein (Figure 1F). Altogether, the results from our flow cytometry experiments are suggestive of coexisting Env populations at the cell surface that can undergo faster or slower internalization. 

### 3.2. Visualizing nNAb-Mediated Env Internalization from the Cell Surface

**Figure 2:**
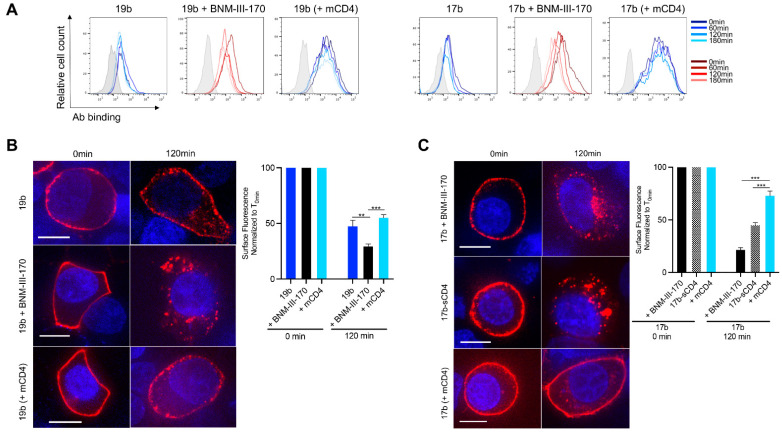
Antibody-induced internalization of Env from the surface of transfected cells. (**A**) Cell surface staining of 293T cells transfected with plasmid encoding JRFL Env alone or together with an expressor of the human CD4 receptor was performed 48-h post-transfection. Ab binding was quantified at 0-, 60-, 120- and 180-min using flow cytometry. Histograms depict representative staining of transfected cells and untransfected (gray) with 17b or 19b Abs. (**B**,**C**) The 293T cells were transfected with a plasmid encoding JRFL Env alone or together with an expressor of the human CD4 receptor and were stained with (**B**) 19b conjugated with Alexa Fluor 647 or (**C**) 17b conjugated with Alexa Fluor 594 for confocal microscopy analyses to visualize internalization. Alternatively, staining with 17b-sCD4 was performed in combination with the goat anti-human IgG Alexa Fluor 594 secondary Ab. (**B**,**C**, Left panels) Images show the localization of antibody–Env complexes at different time points (0 and 120 min). Images represent a single confocal z-section through the middle of the cell; at least 25 cells were imaged per condition, and representative images are shown. Scale bar, 10 μm. (**B**,**C**, Right panels) The remaining cell surface antibody–Env complexes over different time points are expressed as percentages of the surface fluorescence relative to the 0 min time point control. Error bars indicate means ± the SEM. Statistical significance was tested using an unpaired *t* test or a Mann–Whitney U test based on statistical normality (**, *p* < 0.01; ***, *p*  <  0.001). mCD4; membrane-anchored CD4.

We confirmed our observations obtained with infected primary CD4+ T cells (Figure 1) using 293T cells transfected with plasmids encoding the tier 2 JRFL Env alone or together with a human CD4 receptor. Using flow cytometry, we observe an increase in 17b- and 19b-binding to the Env in the presence of CD4mc. Once again, in these conditions, the overall levels of 17b- and 19b-bound Env significantly decreases within 3 h (Figure 2A). However, upon CD4 co-transfection, which allows for greater overall nNAb binding, we observe decreased rates of Env internalization (Figure 2A). To visualize the phenotype of nNAb-induced internalization, we performed confocal microscopy experiments with transfected 293T cells. Cells were incubated 48 h post-transfection with Alexa Fluor-conjugated nNAbs for up to 2 h at 37 °C before fixing and analysis by imaging. In the presence of BNM-III-170, we can visualize a rapid internalization of 19b- and 17b-bound Env from the cell surface compared to the antibody alone (Figure 2B,C). A similar phenotype was observed with Env bound by the 17b-sCD4 chimeric protein (Figure 2C). Similar to the results obtained with the Nef and Vpu deleted virus (Figure 1D), CD4 co-expression did not results in Env internalization, and rather the remaining surface fluorescence was significantly higher (Figure 2B,C). Overall, indicating that, interaction with CD4 in *cis* allows for antibody–Env complexes to remain on the surface for a longer period. These observations confirm previous observations [4], wherein the rates of nNAb-mediated Env internalization were significantly slower upon the co-transfection of the CD4 receptor. Thus, CD4mc or sCD4 speed up Env internalization but membrane-anchored CD4 allows the Env-antibody complexes to remain on the cell surface for a longer period.

### 3.3. Different Membrane Microdomains Could Determine Antibody-Mediated Env Internalization

**Figure 3:**
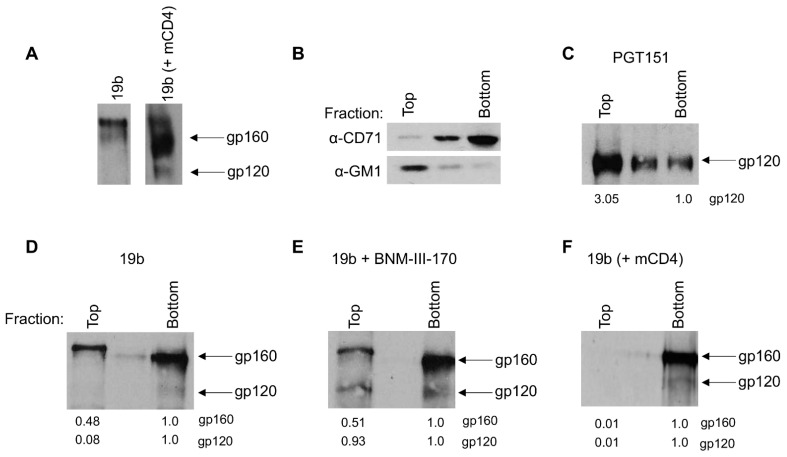
Differential localization of antibody–Env complexes visualized by lipid microdomain fractionation. The 293T cells were transfected with a plasmid encoding JRFL Env alone or together with an expressor of the human CD4 receptor and 48-h post-transfection all cell surface proteins were biotinylated. (**A**) Immunoprecipitation of cell surface biotinylated Env from cells transfected to express the (left) JRFL Env alone or (right) with the human CD4 receptor after incubation with 19b. Further, cell lysates were fractionated on a sucrose density gradient as described in Materials and Methods. (**B**) Equal volumes of individual fractions were resolved by SDS-PAGE, transferred to a nitrocellulose membrane, and probed with HRP-conjugated CTx-B to detect DRM marker ganglioside GM1 or with OKT-9 antibody to detect DSM marker CD71. (**C**–**F**) Immunoprecipitation of cell surface biotinylated Env from individual sucrose gradient fractions using (**C**) PGT151, (**D**) 19b, (**E**) 19b with 5μM BNM-III-170 from cells expressing the JRFL Env only and (**F**) 19b from cells expressing both the JRFL Env and human CD4 receptor. Values represent densities of respective band intensities quantified using ImageJ normalized to the bottom fractions. (**B**–**F**) Representative blots from at least three independent experiments are shown. mCD4; membrane-anchored CD4.

Since we observed striking differences in nNAb-induced internalization rates depending on the presence of membrane-anchored CD4, we sought to determine if this could be due to a difference in the localization of antibody–Env complexes at the plasma membrane. First, we biotinylated Env trimers expressed on the surface of 293T cells, allowed 19b binding, pulled out Env–19b complexes using immunoprecipitation, and resolved surface Env using streptavidin via Western blot. We observed that with cells expressing only the JRFL Env, 19b precipitates out mainly the uncleaved Env (gp160), whereas with cells expressing both the JRFL Env and human CD4, 19b precipitates out both the uncleaved (gp160) and cleaved Env (gp120) (Figure 3A). This is likely due to the conformational flexibility of the uncleaved Env that facilitates its recognition by nNAbs. Env epitopes recognized by these nNAbs are occluded in the cleaved Env [31,32,33], therefore requiring the ‘opening’ of Env by CD4 for their interaction to occur. Of note, and in agreement with previous publications, we observed two bands of distinct molecular weight for the uncleaved gp160 that were previously associated to their glycosylation content (Figure 3A) [7,34].

The Env trimer has been described to be contained in lipid microdomains (commonly known as “lipid rafts”) on the infected cell surface from where budding occurs [35,36,37,38]. These specialized membrane microdomains are enriched with cholesterol and sphingolipids and are reported to play an important role in endocytic processes [39]. To assess the association of rapidly internalized antibody–Env complexes with microdomains, we biochemically isolated lipid microdomains by membrane fractionation using a sucrose gradient [40]. We identified the detergent-resistant membranes (DRMs or “lipid rafts”) using the ganglioside GM1 and the transferrin receptor (CD71) was used to identify the detergent soluble membranes (DSMs) (Figure 3B). Cell surface biotinylated Env trimers complexed with antibodies undergoing fractionation using sucrose gradients were resolved via immunoprecipitation, followed by Western blotting. Using this technique, we observed an enrichment of bNAb-bound (PGT151) cleaved Env (gp120) in the DRM (top) fractions (Figure 3C). Since bNAb-bound Env internalizes rapidly, this initial observation indicates that the Env–antibody complexes that are present in the DRM (top) fractions, and thus, associated with lipid microdomains, undergo rapid internalization. On the other hand, when we used 19b we preferentially detected uncleaved Env (gp160) which was located in both the DSM (bottom) and DRM (top) fractions (Figure 3D). Interestingly, we observed that the slower migrating gp160 band, which was reported to have complex glycans, is enriched in the DRM (top) fractions, while the gp160 band with the lower molecular weight, reported to be composed primarily of oligomannose glycans, appeared in the DSM (bottom) fractions [7,34]. Upon addition of BNM-III-170, the cleaved Env (gp120) appears in the DRM (top) fractions (Figure 3E). The presence of cleaved Env in the fractions that are associated with lipid microdomains upon the addition of BNM-III-170 could explain the rapid Env internalization in the conditions that CD4mc are present. Consistent with our observations that co-expression of CD4 slows down nNAb internalization, we observe an accumulation of the cleaved Env (gp120) localization to the DSM (bottom) fractions (Figure 3F). An enrichment of Ab–Env complexes away from lipid microdomains in the presence of membrane-anchored CD4 could explain the slower antibody-mediated Env internalization seen in these conditions. Thus, cleaved or uncleaved Env-antibody complexes that are present in DSM (bottom) fractions, and do not associate with lipid microdomains, remain on the cell surface for a prolonged period. Our observations suggest that the association of antibody-bound functionally cleaved Env (gp120) with lipid microdomains could influence its accelerated internalization. Future studies need to confirm these observations in a more physiological system with infected primary CD4+ T cells.

Collectively, our results suggest that in addition to the different populations and conformations of Env that coexist on the cell surface, antibody-mediated Env internalization could also be attributed to the localization of Env in discrete microdomains. Further studies to confirm the differential localization of antibody–Env complexes using high-resolution microscopic studies are warranted [41]. Our results could steer the selection of antibody therapeutics against HIV-1 that allow for the Env to remain stable on the surface of infected cells, and thus enhance its recognition by the immune system.

## Data Availability

The data presented in this study are available within this article.
